# Experimental validation and comprehensive analysis of m6A methylation regulators in intervertebral disc degeneration subpopulation classification

**DOI:** 10.1038/s41598-024-58888-w

**Published:** 2024-04-10

**Authors:** Xiaoqian Xu, Lianwei Shen, Yujuan Qu, Danyang Li, Xiaojing Zhao, Hui Wei, Shouwei Yue

**Affiliations:** Rehabilitation Center, Qilu Hospital of Shandong University, Jinan, China

**Keywords:** IVDD, m6A methylation modification, Prediction model, Clustering, Immune infiltration, Computational biology and bioinformatics, Molecular biology, Biomarkers, Diseases

## Abstract

Intervertebral disc degeneration (IVDD) is one of the most prevalent causes of chronic low back pain. The role of m6A methylation modification in disc degeneration (IVDD) remains unclear. We investigated immune-related m6A methylation regulators as IVDD biomarkers through comprehensive analysis and experimental validation of m6A methylation regulators in disc degeneration. The training dataset was downloaded from the GEO database and analysed for differentially expressed m6A methylation regulators and immunological features, the differentially regulators were subsequently validated by a rat IVDD model and RT-qPCR. Further screening of key m6A methylation regulators based on machine learning and LASSO regression analysis. Thereafter, a predictive model based on key m6A methylation regulators was constructed for training sets, which was validated by validation set. IVDD patients were then clustered based on the expression of key m6A regulators, and the expression of key m6A regulators and immune infiltrates between clusters was investigated to determine immune markers in IVDD. Finally, we investigated the potential role of the immune marker in IVDD through enrichment analysis, protein-to-protein network analysis, and molecular prediction. By analysising of the training set, we revealed significant differences in gene expression of five methylation regulators including RBM15, YTHDC1, YTHDF3, HNRNPA2B1 and ALKBH5, while finding characteristic immune infiltration of differentially expressed genes, the result was validated by PCR. We then screen the differential m6A regulators in the training set and identified RBM15 and YTHDC1 as key m6A regulators. We then used RBM15 and YTHDC1 to construct a predictive model for IVDD and successfully validated it in the training set. Next, we clustered IVDD patients based on the expression of RBM15 and YTHDC1 and explored the immune infiltration characteristics between clusters as well as the expression of RBM15 and YTHDC1 in the clusters. YTHDC1 was finally identified as an immune biomarker for IVDD. We finally found that YTHDC1 may influence the immune microenvironment of IVDD through ABL1 and TXK. In summary, our results suggest that YTHDC1 is a potential biomarker for the development of IVDD and may provide new insights for the precise prevention and treatment of IVDD.

## Introduction

Intervertebral disc degeneration (IVDD) is a major cause of chronic low back pain (LBP) and a disabling factor^1,2^. It is caused by imbalances in the anabolic and catabolic processes in the intervertebral disc and is characterized by changes in extracellular matrix (ECM) composition, loss of myeloid nucleus (NP) cells, as well as excessive oxidative stress and inflammation. Furthermore, studies have shown that IVDD is a disease caused by various genetic and environmental factors, with genetic factors taking prominence^3^. IVDD susceptibility has been investigated using a candidate gene method, and researchers have so far identified IVDD-related candidates genes, including genes involved in the structure and function of lumbar discs, genes producing stromal degradation enzymes in lumbar discs, genes associated with the lumbar vertebral bone structure, among others^3,4^. In recent years, the role of RNA modifications in gene regulation has attracted research attention. Among more than 150 RNA modification types identified so far, m6A modification is the most common mRNA modification type in eukaryotes^5^, which regulates metabolic and biological processes in mRNA by targeting transcriptome stability, splicing, translation efficiency, and hat-independent translation. It regulates the development and progression of numerous diseases, including cancer, and neurological, and metabolic diseases^6,7^. Studies on the exact mechanism of the m6A modifying gene in IVDD remain unclear. This study used mechanistic learning combined with LASSO regression analysis to screen key m6A-regulated genes based on RNA expression assay data from IVDD samples in the GENE EXPRESSION OMNIBUS (GEO) database. The key m6A regulatory genes were validated using IVDD rat models and clinical predictive models. IVDD patients were divided into two clusters based on these genes to reveal the immune infiltrating characteristics of different clusters. The resulting key m6A regulatory genes were identified as biomarkers for IVDD. Finally, functional enrichment, protein-to-protein network analysis, and molecular prediction analysis revealed the biological functions and regulatory patterns of key m6A-regulated genes.

## Methods

### Data collection and processing

**Figure 1:**
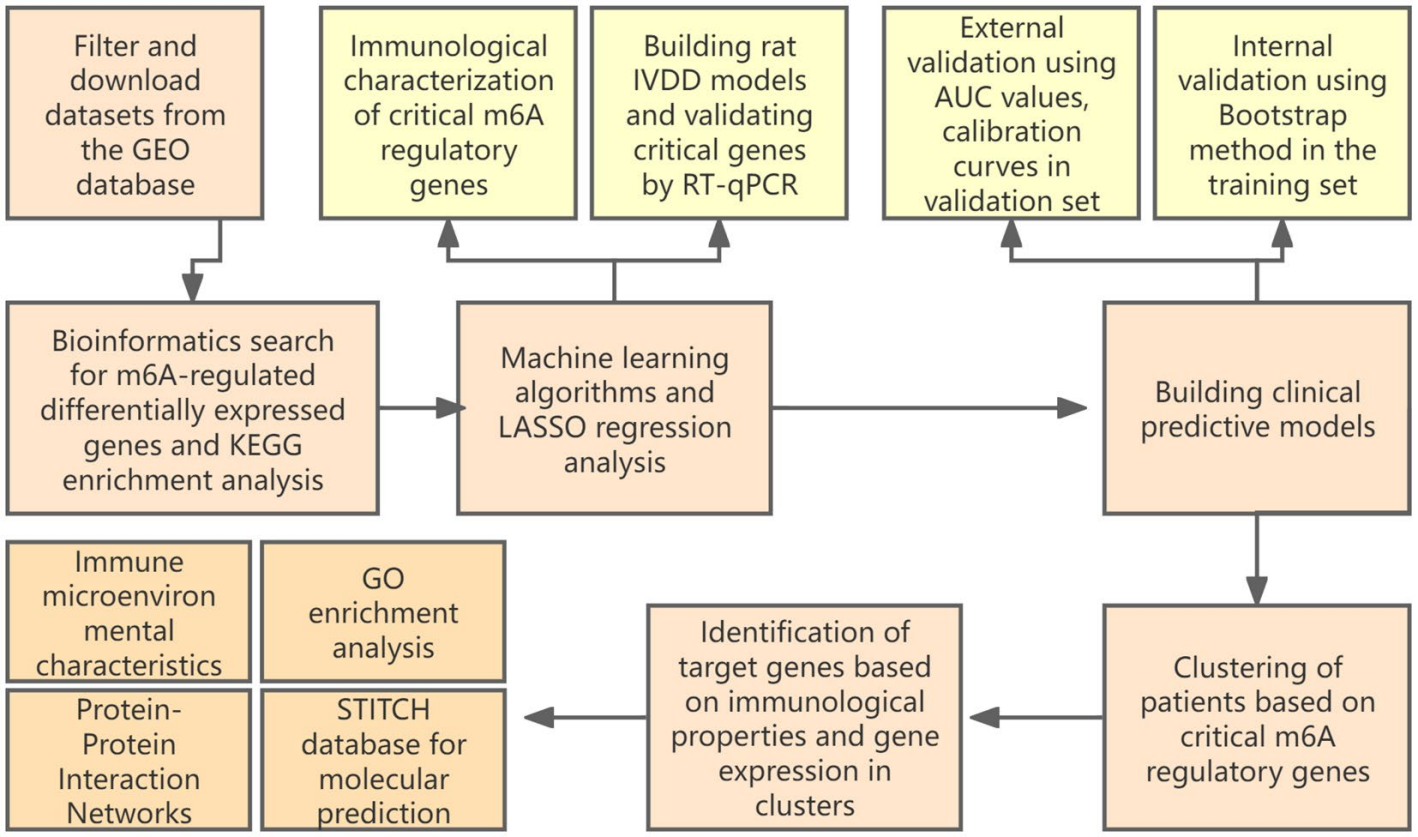
The basic processes involved in the experiment.

The RNA expression profiles and clinical information of datasets GSE15227, GSE23130, GSE147383 and GSE124272 were downloaded from the GEO database, with GSE15227 and GSE23130 as the training set and GSE124272 as the validation set. training set comprising 27 disc tissue samples from IVDD and 11 disc tissue samples from controls. GSE23130 is the “Genome-wide analysis of pain-,” nerve- and neurotrophin-related gene expression in the degenerating human annulus”^8^. GSE15227 is “Prostaglandin E1 and misoprostol increase epidermal growth factor production in 3D-cultured human annulus cells”^9^. The two data sets were from the same research team, and the sample sequencing conditions were consistent, so they could be combined. The validation set included serum RNA samples from 8 IVDD patients and 8 controls. Is “Transcriptome signatures reveal candidate key genes in the whole blood of patients with lumbar disc prolapse”^10^. Validation set is only used as external validation of the model. GSE147383 contains transcriptome sequencing data of 2 cases of degenerative AF and NP and 2 cases of non-degenerative AF and NP. The dataset is from “a spatiotemporal proteomics atlas of human intervertebral discs for exploring ageing and degeneration dynamics”^11^. The expression matrix of the training set and the validation set were normalized by the “normalizbetween-arrays” function of the “limma” package in R language, and the gene probes were annotated with official symbols. The basic processes involved in the experiment are shown in the flow chart (Fig. 1).

### Enrichment analysis of differentially expressed genes

We performed GSEA enrichment analysis on the training set to enrich the biological processes of GO in the training set. According to the grading of phenotypic relevance, the RNA in the training set was divided into high and low expression groups. Then, all differentially expressed genes in the IVDD and control groups were enriched and evaluated using the clusterProfiler package. Reference gene set from c5 molecular signature database (C5.GO.hs.symbols)^12^. A *P* value of less than 0.05 and a false discovery rate (FDR) *P* value of less than 0.20 were considered significant^13^.

### The functional role of m6A regulated genes on IVDD, screening of m6A differentially expressed genes and characterization of immune infiltration in training sets

Based on the current research literature on m6A, 26 m6A-regulated genes were included as subjects, including METTL3, METTL14, METTL16, WTAP, VIRMA, ZC3H13, RBM15, RBM15B, CBLL1, YTHDC1, YTHDC2, YTHDF1, YTHDF2, YTHDF3, HNRNPC, FMR1, LRPPRC, HNRNPA2B1, IGFBP1, IGFBP2, IGFBP3, RBMX, ELAVL1, IGF2BP1, FTO, ALKBH5^14^. After viewing the expression of the above genes in IVDD, KEGG enrichment analysis of m6A regulated genes in IVDD was performed. After that, the expression of m6A-related genes in each sample was extracted using the “limma” package in the R language. The Wilcoxon test was then used to detect differences between the training set of IVDD patients and the control group in the above m6A-related gene expression model, and the differences were screened at *P* < 0.05. The differentially expressed m6A-associated genes in the training set were screened at *P* < 0.05. A total of 22 immune cells identified in previous studies were selected for this study^15,16^ including B cells naïve, B Cells memory, Plasma cells, T cells CD8, T cells CD4 naïve, T cells CD4 memory resting, T cells CD4 memory activated, T cells follicular helper, T cells regulatory (Tregs), T cells gamma delta, NK cells resting, NK cells activated, Monocytes, Macrophages M0, Macrophages M1, Macrophages M2, Dendritic cells resting, Dendritic cells activated, Mast cells resting, Mast cells activated, Eosinophils and Neutrophils. The immune cell content of each sample in the training set was analyzed in R using the “CIBERSORT” function from the “preprocessCore” package, and then the “stat_compare_means” function from the “ggplot” package was used to perform a statistical analysis of the immune cell content of the samples in the training set.

### Establishment of IVDD model in rats and histological examination

Twelve healthy clean grade adult male Wistar rats with an average weight of 220 ± 20 g were purchased from Jinan Pengyue Experimental Animal Breeding Co. We used as few rats as possible while meeting statistical requirements. The rats were randomly and equally divided into a blank group and a puncture group of six rats each. For the experimental group, we anesthetized Wistar rats with isoflurane and first identified the caudal intervertebral discs (Co7/8, Co8/9) by palpation; we then inserted a sterile 17-gauge needle into the middle of the disc at a depth of 5 mm and rotated it 360 degrees to disrupt the disc fibrous annulus to construct the IVDD model; the control group was left untreated. The rats were executed 4 weeks after modeling, and the nucleus pulposus and surrounding fibrous annulus tissue were removed from the caudal intervertebral discs (Co7/8, Co8/9) of the rats for subsequent experiments^17^. Rat tail intervertebral disc samples were fixed in 4% neutral-buffered paraformaldehyde, embedded in paraffin, and sectioned continuously (5-μm thick). The Saffron-O Stain was performed according to the manufacturer’s instructions (Solarbio, Beijing, China).

### Real-time quantitative fluorescence polymerase chain reaction PCR (RT-qPCR) for the detection of m6A differentially expressed genes in intervertebral disc tissue in rats with IVDD model.

Total RNA was isolated from the nucleus pulposus and surrounding fibrous ring tissue using an RNA extraction kit (Fadagen Biotechnology, Shanghai). RNA was converted to cDNA following the instructions of the manufacturer's protocol (vazyme, Nanjing), and the cDNA was used as a template for RT-qPCR, using GAPDH as an endogenous control to normalize differences in mRNA detection. Melting curves were created after cycle completion to confirm the absence of non-specific products. We quantified mRNA levels by GAPDH Ct normalized Ct (cycle threshold) values and analyzed them using the 2ΔΔCT method. The primer sequences used are described in the supplementary material (Biosune, Shanghai).

### Screening of m6A key regulatory genes, and their expression differences in AF and NP

Machine learning algorithm models (random forest, support vector machine) and LASSO regression analysis were selected for screening m6A key regulatory genes. The machine learning algorithm model was used to screen the m6A key regulatory genes: the residual values, the reverse residual distribution, and the area under the ROC curve of the m6A differentially expressed genes were calculated using the “randomForest” and “pROC” packages in the R language. LASSO regression analysis: LASSO regression analysis is used to select variables by reducing the coefficient weights of variables that are not relevant to the outcome to zero^18^. We performed LASSO regression analysis using the “glmnet” package to calculate coefficient scores for m6A differentially expressed genes, and screened key m6A regulatory genes with coefficients scores < 0.05. Next, we identified the key m6A regulatory genes by taking the intersection of the above machine learning algorithm and the key m6A regulatory genes screened by LASSO regression analysis. Finally, we looked at the expression differences of key regulatory genes in AF and NP based on the dataset GSE147383, and the analysis method was as described above.

### Construction and validation of clinical prediction models based on m6A key regulatory genes

The “datadist” function in the rms package was used to package the expression of m6A key regulatory genes in the training set, and then the “lrm” function was used to fit the clinical prediction model. The clinical prediction models were evaluated using the C-index for discrimination, calibration curves for concordance, and decision curves for model patient benefit^19^.

### Clustering of IVDD patients in the training set based on m6A key regulatory genes and looking at the immune microenvironmental characteristics of the clusters

Cluster analysis was used to cluster the IVDD patients in the training set based on the m6A key regulatory gene: The “ConsensusClusterPlus” function in the “ConsensusClusterPlus” package was used to cluster the IVDD patients in the training set eight times based on the expression of the m6A key regulatory gene, and the IVDD patients were divided into eight different clusters. The “Rtsne” package is used to display the distribution of samples in different clusters. The best clusters are selected based on the criteria of high intra-cluster correlation and low inter-cluster correlation. The clusters are viewed in R using Principal Component Analysis (PCA). Expression of key m6A regulatory genes under this sub-cluster was compared in R using the Kruskal–Wallis test and visualized using heat maps with box plots. Immune microenvironment characterization of clusters: single sample gene set enrichment analysis (ssGSEA) using the 'gsva' package in R to analyze the immune infiltration of samples and genes. Based on previous studies^20^, 23 types of immune microenvironment cells were selected for this study, including Activated. B cell、Activated.CD4.T.cell、Activated.CD8.T.cell、Activated.dendritic.cell、CD56bright.natural.killer.cell, CD56dim.natural.killer.cell、Eosinophil、Gamma.delta.T.cell、Immature.B.cell、Immature.dendritic.cell、MDSC、Macrophage、Mast. cell、Monocyte、Natural.killer.T.cell、Natural.killer.cell、Neutrophil、Plasmacytoid.dendritic.cell、Regulatory.T.cell、T.follicular.helper.cell、Type.1.T.helper.cell、Type.17.T.helper.cell and Type.2.T.helper.cell. Subsequently, the expression of infiltrating immune cells in the control and patient groups was compared in R using the Kruskal–Wallis test. A correlation heat map was created using the “pheatmap” package to show the correlation between key m6A-regulated genes and the above immune cells under different sub-clusters.

### Identification of IVDD biomarkers, immune microenvironment characterization, enrichment analysis, protein interaction network analysis, and molecular prediction

Identification of IVDD biomarkers: Identification of biomarkers of IVDD based on the expression heat map of key regulatory genes under optimal clustering and correlation between key m6A regulatory genes under optimal clustering and the above immune cells. Immune microenvironment characterization: Based on the expression of this biomarker, IVDD patients in the training set were divided into high and low-expression groups, and the expression of infiltrating immune cells in the high and low-expression groups was compared using the Kruskal–Wallis test. Enrichment analysis: GO enrichment analysis of this biomarker was performed in R language. Protein Interaction Network Analysis: The STRING database is dedicated to the construction of protein association networks for organisms and can be used to integrate all known and predicted associations between proteins, including physical interactions and functional associations^21^. Molecular prediction: The STITCH (Search Tool for Interactions of Chemicals) database is an online resource focused on molecular interactions and networked drug discovery, designed to integrate multiple data on biologically active compounds, proteins, genes, metabolic pathways, etc. and digitally present the interaction and signaling networks between them^22^.

### Ethics statement

All experimental procedures have been reviewed andapproved by the Animal Ethical and Welfare Committee of Shandong University. All methods were carried out in accordance with relevant guidelines and regulations. All methods are reported in accordance with ARRIVE guidelines (https://arriveguidelines.org).

## Results

### GSEA enrichment analysis

**Figure 2:**
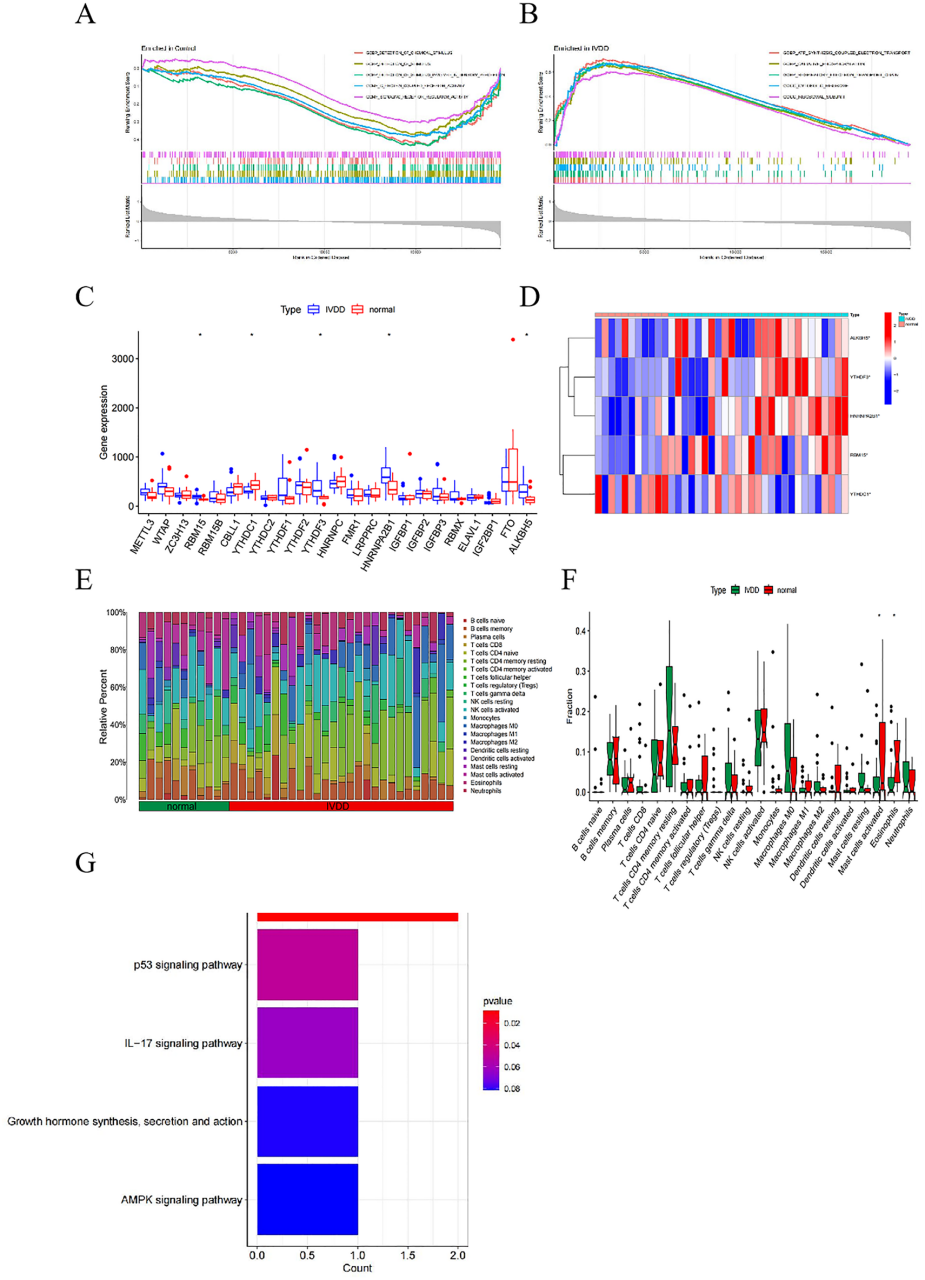
Differentially expressed m6A genes in IVDD and their immunological characteristics. (**A**) Results of GSEA analysis of control samples in the training set. (**B**) Results of GSEA analysis of IVDD samples in the training set. (**C**) Boxplot of m6A gene expression comparison between Normal and IVDD groups. (**D**) Heat map of five m6A differentially expressed genes in Normal and IVDD groups. Red represents high expression and blue represents low expression. (**E**) Content of 22 immune cell species in patients in Normal versus IVDD groups. (**F**) Differences in the content of 22 immune cells in patients in the Normal versus IVDD groups. *P < 0.05, **P < 0.01, ***P < 0.001. (**G**) Barplot shows KEGG pathway enrichment results of m6A differentially expressed genes in IVDD.

GSEA was used to identify pathways that differed significantly between IVDD samples and control samples in the training set. The results showed that, control samples were enriched for detection of chemical stimulus, detection of stimulus, detection of gastrointestinal peptides for stimulus involved in sensory perception, protein-coupled receptor activity, and signal receptor regulator activity (Fig. 2A). IVDD samples were enriched in ATP synthesis couples electron transport, oxidative phosphorylation, respiratory electron transport chain, ribosomes and ribosomal subunits (Fig. 2B).

### Screening for m6A differentially expressed genes in IVDD and associated immune infiltration characterization, and the functional roles of these m6A differentially expressed genes genes

By analyzing the training set, we found that 23 M6A-related genes, including METTL3, WTAP, ZC3H13, RBM15, RBM15B, CBLL1, YTHDC1, YTHDC2, YTHDF1, YTHDF2, YTHDF3, HNRNPC, FMR1, LRPPRC, HNRNPA2B1, IGFBP1, IGFBP2, IGFBP3, RBMX, ELAVL1, IGF2BP1, FTO and ALKBH5, were expressed in IVDD. Statistical analysis revealed significant differences in the expression of five genes, including YTHDC1, RBM15, HNRNPA2B1, YTHDF3, and ALKBH5, among which YTHDC1 was significantly down-regulated in IVDD patients, whereas RBM15, HNRNPA2B1, YTHDF3 and ALKBH5 were significantly up-regulated (Fig. 2C,D). Interestingly, we found that HNRNPA2B1 was also considered as one of the key genes of IVDD in the biological letter published by Tao T. et al., and was related to immune cell infiltration during the development of IVDD^13^. The TRRUST database shows that the transcription factor MYC can activate the expression of HNRNPA2B and regulate the metabolic phenotype^23^. While breast cancer susceptibility gene 1 can inhibit the expression of HNRNPA2B, thereby regulating RNA processing^24^. The immune cell content of each sample in the Training set is shown in (Fig. 2E), while we found significant differences in Mast cells activated and Eosinophils between the IVDD and normal groups (Fig. 2F). In addition, KEGG enrichment analysis showed that the above genes and Spliceosome, p53 signaling pathway, IL-17 signaling pathway, Growth hormone synthesis, secretion and action, and AMPK signaling pathway (Fig. 2G)^25–27^.

### Expression profiles of m6A differentially expressed genes in IVDD models

**Figure 3:**
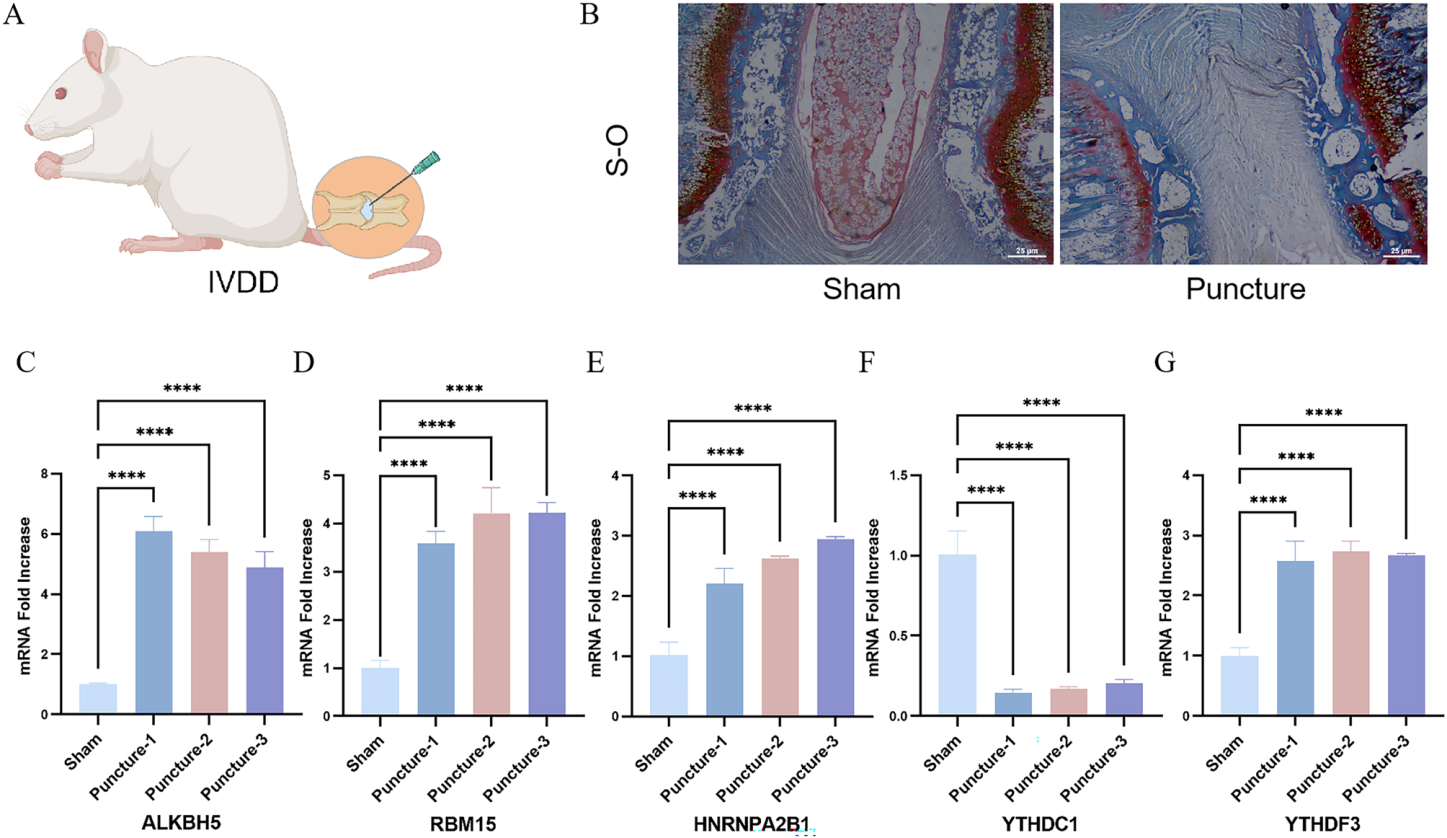
Establishment of IVDD animal model and RT-qPCR (**A**) Schematic diagram of the puncture-induced IVDD model. (**B**) Safranin-O staining of normal and IVDD rats. (**C**–**G**) Expression levels of RBM15, YTHDF3, HNRNPA2B1, ALKBH5, YTHDC1 in caudal disc tissues of Normal and IVDD groups were detected by RT-qPCR. ****P < 0.0001.

A rat IVDD model was constructed to validate the expression of the above m6A differentially expressed genes in IVDD, and the Safranin-O staining indicated that the IVDD model was successfully established in rats (Fig. 3A,B). At the time of sampling, we found that the rats in the IVDD group had less nucleus pulposus tissue in the caudal intervertebral disc compared with the normal group, with greyish-white color. The five m6A regulatory genes screened were validated by RT-qPCR. The results showed that the expression of RBM15, YTHDF3, HNRNPA2B1, and ALKBH5 was significantly up-regulated in the IVDD group compared to the normal group, whereas YTHDC1 expression was significantly down-regulated in the IVDD group compared to the normal group, which was consistent with the results of previous difference analysis (Fig. 3C–G).

### Screening of m6A key regulatory genes based on multiple algorithms, and validating the expression of them in AF and NP

**Figure 4:**
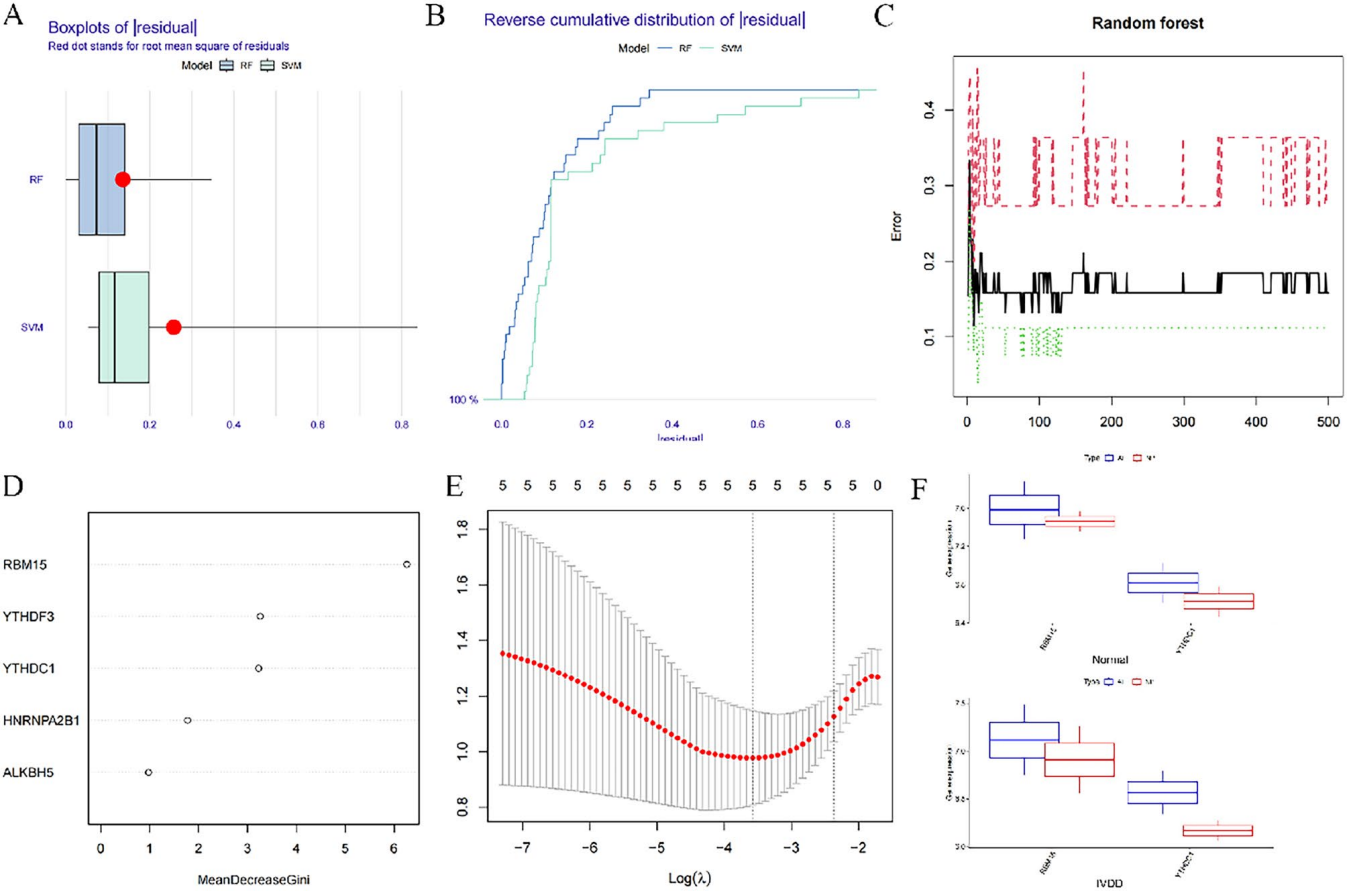
Screening of m6A key regulatory genes. (**A**) Box line plots of residuals in RF and SVM. (**B**) Inverse cumulative distribution of residuals in RF and SVM. (**C**) Random forest screening of deg. (**D**) Screening of candidate m6a regulatory genes by RF. (**E**) Screening of m6a regulatory genes by lasso regression. (**F**) Box plots represent the expression of RBM15 and YTHDC1 in AF and NP in nondegenerative and degenerative disc tissues.

We compared the two machine learning algorithms by calculating residual values, reverse residual distribution plots, and the area under the AUC curve. The RF algorithm outperformed SVM in the expression of residual values and the area under the AUC curve (Fig. 4A, B); therefore, the RF algorithm was selected for screening m6A key regulatory genes. According to the RF results, the smallest the algorithm is observed when there are 198 trees (Fig. 4C). Currently, there is no standardized approach for determining importance scores in RF algorithms; however, these scores typically exceed a value of 2^28^.The genes RBM15, YTHDC1, and YTHDF3 with importance scores greater than 2 were selected as the machine learning algorithm to derive the m6A key regulatory genes (Fig. 4D). The LASSO analysis of the training set was performed in R using the “glmnet” package, and the LASSO path diagram in Fig. 4E shows the presence of the horizontal coordinates “lambda.min” and “lambda.1se”, the coefficients scores of the five m6A differential expressions, including RBM15, YTHDC1, YTHDF3, HNRNPA2B1, and ALKBH5, were calculated. The coefficients scores < 0.05 were then considered as significant differences, and RBM15 and YTHDC1 were screened as key m6A regulatory genes. Finally, the intersection of the m6A key regulatory genes screened by the machine learning algorithm and the LASSO regression analysis was taken to arrive at the key m6A regulatory genes: RBM15 and YTHDC1. We found that the expression of RBM15 and YTHDC1 was higher in AF than in NP in both degenerative and non-degenerative disc tissues (Fig. 4F).

### Construction and validation of clinical prediction models based on RBM15 and YTHDC1

**Figure 5:**
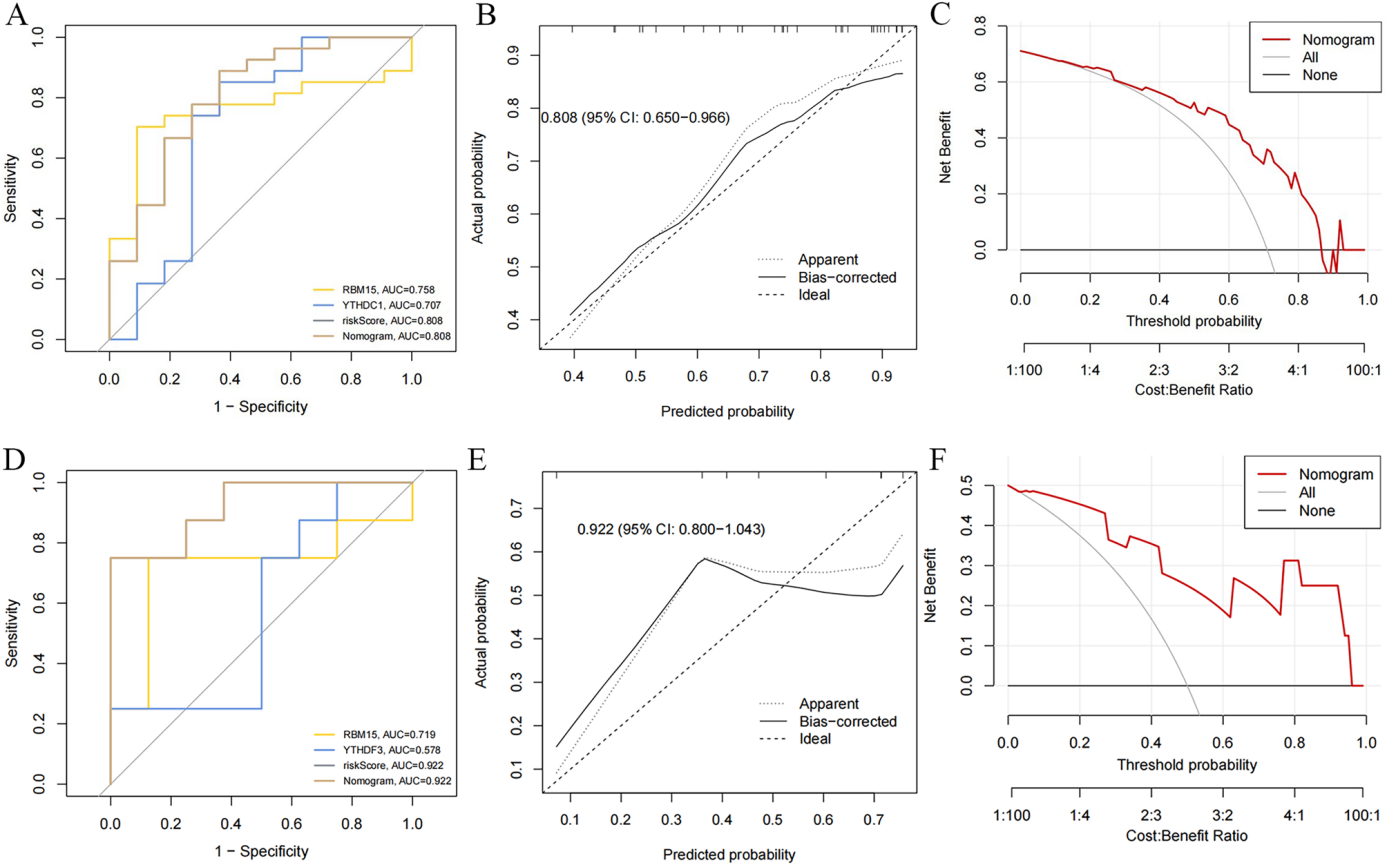
Construction and validation of clinical prediction model based on RBM15, YTHDC1 (**A**) C_index of clinical prediction model. (**B**) Calibration curve of clinical prediction model. (**C**) Decision curve of clinical prediction model (DCA). (**D**) C_index of RBM15, YTHDC1 in validation set. (**E**) Calibration curve of RBM15, YTHDC1 in validation set. (**F**) Decision curve of RBM15, YTHDC1 in validation set (DCA).

A clinical prediction model based on the expression of RBM15 and YTHDC1 was constructed in the TRAINING SET to assess the risk association of RBM15, YTHDC1, and IVDD. The C-index of this clinical prediction model was 0.808 (95% CI 0.650–0.966) (Fig. 5A). The calibration curve based on RBM15, YTHDC1 plotted in the TRAINING SET illustrates the high calibration of the model (Fig. 5B). The decision curve (DCA) of the model was higher than both the None and ALL reference lines in the interval of 0.2–0.85 (Fig. 5C). The area under the ROC curve of RBM15, YTHDC1 in the validation set was 0.922 (95% CI 0.800–1.043) (Fig. 5D); the calibration curve plotted for RBM15, YTHDC1 in the validation set (Fig. 5E). The decision curves (DCA) for RBM15, YTHDC1 in the validation set were higher than both the None and ALL reference lines in the interval of 0.04–0.95 (Fig. 5F).

### Clustering analysis of IVDD patients based on RBM15, YTHDC1

**Figure 6:**
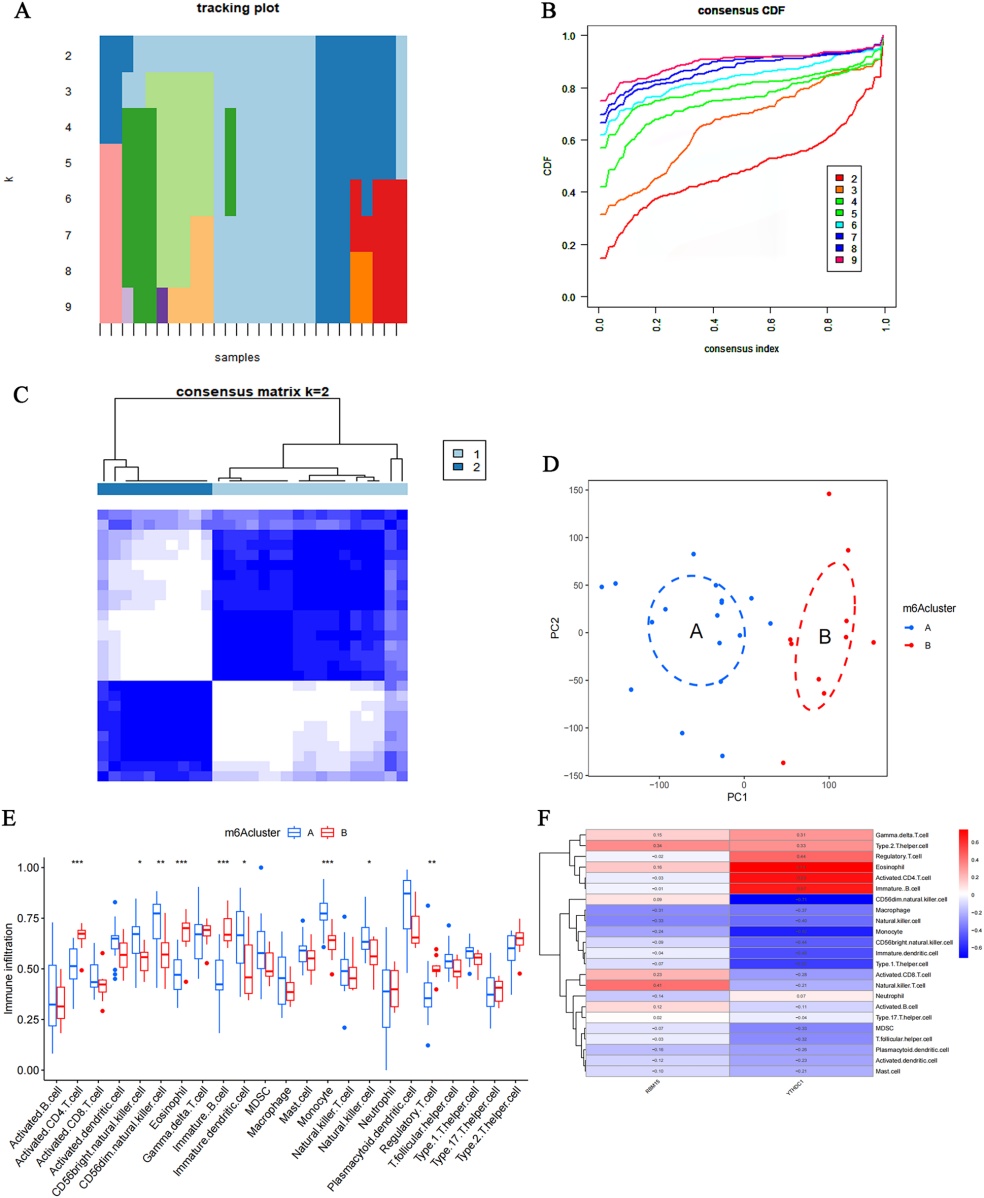
Cluster analysis of IVDD patients based on RBM15, YTHDC1. (**A**,**B**) Eight sub-clusters of RBM15, YTHDC1 for IVDD patients. (**C**) Correlation of patients between groups when the consensus clustering matrix k = 2. (**D**) PCA analysis at optimal clustering. Groups A and B represent two categories of IVDD patients based on the expression of RBM15, YTHDC1. (**E**) Expression of 23 immune cells based on optimal clustering. (**F**) Heat map of the correlation between immune cell infiltration of RBM15, YTHDC1. Red indicates positive correlation, blue indicates negative correlation.

Based on RBM15, YTHDC1, we divided IVDD patients into 8 clusters (Fig. 6A,B). Based on the criteria of high intra-cluster correlation and low inter-cluster correlation, patients were selected to be divided into 2 clusters as the optimal clusters for this study (Fig. 6C). Principal component analysis (PCA) revealed that the above clustering analysis method could precisely classify IVDD patients into two clusters (Fig. 6D). Therefore, the clustering of IVDD patients based on RBM15 and YTHDC1 in this study was accurate. Our enumeration analysis of the immune cell statistic between the 2 clusters found Activated.CD4.T.cell, CD56bright.natural.killer.cell, CD56dim.natural.killer.cell, Eosinophil, Immature.B.cell, Immature, Immature. dendritic cell, Monocyte, Natural.killer.cell, and Regulatory.T.cell were significantly different for a total of nine immune cell infiltration types (Fig. 6E). Meanwhile, we calculated the correlation between RBM15, YTHDC1, and immune cell infiltration (Fig. 6F), in which YTHDC1 had the most significant correlation, with a maximum positive correlation coefficient of 0.74 and a maximum negative correlation coefficient of -0.71. Therefore, YTHDC1 may play a key role in immune cell infiltration.

### Identification of IVDD biomarkers, immune microenvironment characterization, enrichment analysis, protein interaction network analysis, and molecular prediction

**Figure 7:**
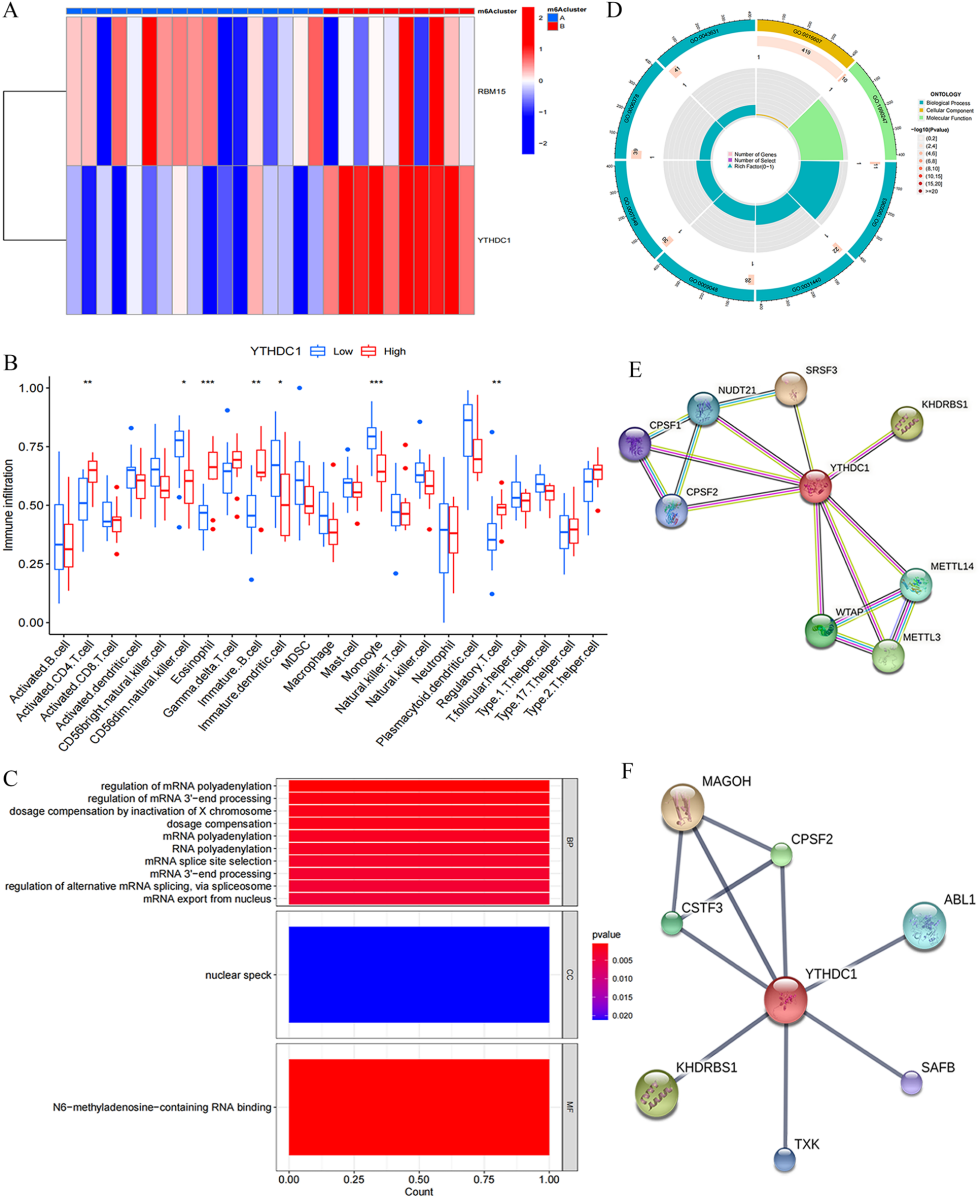
Identification of IVDD biomarkers, immune micro-environment characterization, enrichment analysis, protein interaction network analysis and molecular prediction. (**A**) Heat map of expression of RBM15, YTHDC1 under two subclusters Red indicates positive correlation, blue indicates negative correlation. (**B**) Expression of 23 immune cells under YTHDC1-based expression grouping *P < 0.05, **P < 0.01, ***P < 0.001. (**C**,**D**) GO enrichment analysis of YTHDC1. (**E**) YTHDC1-based protein interactions network analysis. (**F**) Molecular prediction based on YTHDC1.

We evaluated the differential expression of YTHDC1 and RBM15 under the 2 subclusters and discovered that YTHDC1 was significantly, and differentially expressed under the 2 sub-clusters (Fig. 7A), which in combination with the results of the previous section caused the highest correlation of YTHDC1 in immune cells; thus, we identified YTHDC1 as a biomarker for IVDD. Based on the expression of YTHDC1, statistical differences were noted in immune infiltrating cells in seven immune microenvironments: activated.CD4.T.cell, CD56dim.natural.killer.cell, Eosinophil, Immature B-cell, Immature dendritic cell, Monocyte, Regulatory.T.cell (Fig. 7B). To explore the biological functional characteristics of YTHDC1 under the expression pattern, GO enrichment analysis of YTHDC1 was performed in R language (Fig. 7C). YTHDC1 was significantly enriched in biological processes (BP) regulation of mRNA polyadenylation; in cellular components (CC) nuclear speck was significantly enriched; and in molecular functions (MF) N6-methyladenosine-containing RNA binding was significantly enriched. The GO circle diagram shows the number of patients enriched in different GO pathways (Fig. 7D). We performed protein interaction network analysis in the STRING database indexed by YTHDC1, setting the minimum required interaction score to the highest confidence (0.900), and obtained the relevant key molecules as CPSF2, CPSF1, NUDT21, SRSF3 KHDRBS1, WTAP, METTL3, METTL4 (Fig. 7E). Molecular predictions were performed in the STITCH database using YTHDC1 as the index, setting the minimum required interaction score to the highest confidence (0.900); we obtained the relevant key molecules as CPSF2, MAGOH, KHDRBS1, ABL1, TXK SAFB, and CSTF3 (Fig. 7F).

## Discussion

This work found that the m6A methylation regulatory gene YTHDC1 may modulate IVDD development by targeting the immune microenvironment. Low back pain (LBP) is an extremely common health problem, affecting more than 80% of the general population. Lumbar disc disorders (LDDs), including symptomatic lumbar disc degeneration (LDDg) and lumbar disc herniation (LDH), are the leading causes of low back pain^29^.

Disc degeneration is a polygenic disease influenced by environmental and genetic factors^30^. Many twins and families with a genetic predisposition to early-onset sciatica and LDH confirm that genetic factors largely contribute to disc degeneration^29^. Moreover, researchers believe that the phenomenon may occur because of defects in incomplete episomal dominant genes causing alterations in collagen structure, which is an important cause of degenerative disc degeneration^31^. Variants in the matrix metalloproteinase-3 gene are also associated with disc degeneration among the elderly^32^. Research has shown that ozone stimulates the immune system to resorb herniated portions of the nucleus pulposus and may promote the progression of intervertebral degenerative lesions as an epigenetic modulator. This suggests that epigenetics have considerable research potential in IVDD. However, the mechanisms of epigenetic modifications, represented by m6A methylation modifications in IVDD remain understudied.

The present study aimed at exploring whether m6A-regulated genes play a role in the immune microenvironment of IVDD and their possible mechanisms. First, we downloaded the datasets GSE15227 and GSE23130 from the GEO database as the training set and GSE124272 as the validation set. Next, we used the intervertebral disc samples of the IVDD group and the control group from the GEO database as the training set, and found that 23 m6A related genes were expressed in IVDD. KEGG enrichment analysis showed that the above m6A regulated genes affected Spliceosome of IVDD, p53 signaling pathway, IL-17 signaling pathway, Growth hormone synthesis, secretion and action, and AMPK signaling pathway. Spliceosome is highly related to degenerative diseases and immune regulation^33^. p53 signaling pathway, IL-17 signaling pathway, Growth hormone synthesis, secretion and action, and AMPK signaling pathway have been proved to be highly related to the occurrence and development of IVDD^34–37^.

Thereafter, we examined the immune cell characteristics of the samples in the training set using bioinformatics techniques and discovered that activated Mast cells and overexpressed Eosinophils in IVDD patients were statistically different. This is consistent with the finding of Kaneyama et al.^38^ that immune cells bind to the nucleus pulposus cells to generate an immune response, causing apoptosis of the nucleus pulposus cells after compromising the physiological barrier of the disc cells. Furthermore, five genes, including YTHDC1, RBM15, HNRNPA2B1, YTHDF3, and ALKBH5 were significantly differentially expressed in IVDD patients, unlike the normal controls in the training set. Subsequently, we collected intervertebral disc samples from IVDD and control rats to verify the confidence of differentially expressed regulators. In recent years, integrated algorithms, represented by RF have shown significant advantages over single classifiers, represented by SVM, in terms of processing highly nonlinearly correlated data, robustness and tuning simplicity of noiseand efficient parallel processing^39,40^. Contemporary research has shown that integrated algorithms have an important role in classifying imaging data for neurological diseases^41^. LASSO regression analysis has been extensively adopted in basic medical research and can be completed to screen key genes by computing the risk scores of genes^42,43^. Furthermore, machine learning and LASSO regression analysis were selected to screen out m6A key regulatory genes. The intersection of the two was taken for further analysis, and we eventually concluded that RBM15 and YTHDC1 are the key m6A regulatory genes based on the importance score of RF and coefficients score of LASSO regression analysis. To verify the reliability of RBM15 and YTHDC1 as key m6A regulatory genes for IVDD, we used RBM15 and YTHDC1 to construct a clinical prediction model, which was validated using serum sequencing data from the IVDD group and the control group in the GEO database. This study adopted a diagnostic model to calculate the risk probability of RBM15 and YTHDC1 in IVDD, which can be used for genetic diagnosis of IVDD^43^. After constructing the clinical prediction model, it is necessary to assess model performance using various model performance metrics^44^; it is also important to perform external validation. Of note, external validation uses a dataset temporally and geographically different from the training set. Therefore, external validation is a process of repeating the development of the model in the validation set^45,46^. Calibration and discrimination are the most fundamental and important metrics in the training and validation sets^47^. Calibration is the extent to which the predicted risk of a predictor agrees with the observed results, and is assessed by plotting a graphical representation of the calibration curve^48^.

In this work, the calibration degree curves of the clinical prediction models and external validation predicted consistent risk curves with the observed outcome curves. This indicates that RBM15 and YTHDC1 have high predictive power in both the training and validation sets. The discrimination is the capacity of the model to distinguish between true positives and true negatives; it is assessed using the C- index, which is equivalent to the area under the curve of the subject's working characteristics, with higher values representing a model that can distinguish between true positives and true negatives^49^. The C -index for RBM15 and YTHDC1 in the training and validation sets was 0.808 and 0.922, respectively. This indicates that the predictive ability based on clinical prediction models based on RBM15 and YTHDC1 can accurately distinguish between patients with IVDD disease and those with the non-IVDD disease. At the same time, decision curve analysis was introduced as a complement to the discrimination and calibration. Notably, decision curve analysis is a method to determine whether the use of predictive models in the clinic to inform decisions is more beneficial than detrimental^50^. Here, the decision curves of RBM15 and YTHDC1 on both the training and validation sets were favorable to patients over most of the range. This indicates that the use of RBM15 and YTHDC1 is clinically plausible for risk prediction in IVDD. As a type of m6A methyltransferase, RBM15 can bind to the m6A complex, thereby recruiting the m6A complex to a specific RNA binding site^51^.

The enzyme can be used as a prognostic marker for a variety of tumours and there is a diversity of modes of action in tumours^52^. For instance, the enzyme is an independent protective prognostic factor in uveal melanoma^53^ and an independent risk prognostic factor in pancreatic adenocarcinoma, where its knockdown inhibits the proliferation of pancreatic adenocarcinoma cells^54^. Members of the YTH structural domain family constitute a large class of m6A readers, and YTHDC1 is associated with RNA splicing^55^ and the export of m6A-modified RNA from the nucleus to the cytoplasmic export^56^. In pancreatic cancer, YTHDC1 hypermutation suggests a poor outcome in pancreatic cancer^57^. On the other hand, YTHDC1 may act as an independent prognostic gene in lung squamous carcinoma^58^. In non-neoplastic diseases, high YTHDC1 expression can result in the upregulation of mitochondrial autophagy-related genes (PINK and PRKN) and increased myocardial energy expenditure in cardiomyocytes with hypertrophic cardiomyopathy, hence may be a potential biomarker for hypertrophic cardiomyopathy^59^.

Afterward, a cluster analysis was performed using a cluster clustering algorithm based on the expression of both in IVDD patients in the training set^60^ to assess the importance of RBM15 and YTHDC1 in IVDD and their role in the immune microenvironment. IVDD patients were divided into 2 clusters based on the criteria of high correlation and low correlation between groups. Principal component analysis was performed to assess the confidence level of this cluster. Of note, principal component analysis is used to define the underlying structure of the data based on correlations between variables^61^. In this study, groups A and B were used to identify the 2 sub-clusters and the 2 clusters can distinguish patients based on the PCA plots. From the statistical map of the immune microenvironment, RBM15 and YTHDC1 affect cells in the immune microenvironment of IVDD and play an important role in the immune microenvironment. Existing studies indicate that immune cells and their inflammatory factors can further accelerate the catabolic environment via defective infiltration in cartilage endplates and annulus fibrosus fissures during intervertebral disc degeneration^62^. Immunotherapy targeting immune cells may be a novel strategy to alleviate IVDD^63^. Meanwhile, genes associated with immune cells may become biomarkers of IVDD^64^. We reviewed the correlation heat map of immune cell infiltration of RBM15 and YTHDC1 as well as the expression heat map of RBM15 and YTHDC1 under two sub-clusters to further assess the importance of RBM15 and YTHDC1 in IVDD. Consequently, we ascertained that YTHDC1 is an important immune-related biomarker in IVDD.

Finally, we performed GO enrichment analysis of YTHDC1 and found that YTHDC1 was enriched in N6-methyladenosine-containing RNA binding at molecular function (MF), which is consistent with We findings. This is consistent with the finding of Xiao et al. that YTHDC1 binds to mRNA and then regulates splicing factors to target mRNA binding regions. This molecule is enriched in the nuclear speck on the Cell Component (CC) and existing studies on the role of YTHDC1 in tumors indicate that YTHDC1 maintains the structure and function of the nuclear speck, which regulate tumor cell genesis and metastasis^65,66^. YTHDC1 is enriched in Biological Process (BP) to regulate downstream mRNA processes including splicing, 3'-end processing, and polyadenylation, with splicing and 3'-end processing discussed above, whereas polyadenylation refers to the process of cleavage of pre-mRNA at the poly(A) site and addition of the poly(A) tail, which is necessary for mRNA formation^67^; and YTHDC1 can regulate polyadenylation by interacting with molecules upstream of polyadenylation or its polyadenylation^68^. Our protein interaction analysis and molecular prediction of YTHDC1 in the STRING and STITCH databases revealed that YTHDC1 is associated with various RNA adenylation-specific subunits on the protein interaction network, including with CPSF1, CPSF2, and NUDT21, besides other m6A modifying enzymes, which is consistent with the results of the GO enrichment analysis. YTHDC1 was also associated with KHDRBS1, suggesting that protein phosphorylation may also play a role in IVDD. Our molecular prediction of YTHDC1 revealed its relationship with ABL1 and TXK tyrosine kinase, besides similar adenylation-specific subunits and splicing-related factors as in the protein interaction network analysis. ABL1 positively correlates with inflammatory cytokine levels and inflammatory pathway activation, Additional studies indicate that ABL1 inhibition suppresses phosphorylation of IκBα and p38 as well as attenuates inflammation levels^69^. This triggers speculation that YTHDC1 may participate in the development of IVDD through its relationship with the ABL1/NF-kB/P38 pathway. txK tyrosine kinase, a non-receptor tyrosine kinase of the Tec family, specifically enhances the phospholipase C (PLC)-γ1-mediated calcium signaling pathway when the T cell antigen receptor (TCR) is involved^70^. Its expression is closely associated with Th1/Th0 cell development and is significantly involved in the cellular production of IFN-γ through Th1 cell-specific positive transcriptional regulation of the IFN-γ gene^71^, suggesting that YTHDC1 may also affect the immune microenvironment of IVDD by interacting with TXK tyrosine kinase. These results suggest that YTHDC1 may play an important role in the immune microenvironment of IVDD. We verified that YTHDC1 may be an immune biomarker of IVDD and might affect the immune microenvironment of IVDD through ABL1 and TXK, in turn affecting the development and progression of IVDD.

We confirmed that YTHDC1 may be an immune biomarker of IVDD and might affect the immune microenvironment of IVDD through ABL1 and TXK, hence the development and progression of IVDD. This work has limitations. First, although we innovatively identified YTHDC1 as an immune marker of IVDD and its possible relationship with ABL1 and TXK to influence the immune microenvironment of IVDD, we did not validate the expression of these molecules in the clinical setting. Secondly, the absence of the above molecules for more in-depth interaction mechanisms is important and can be comprehensively analyzed in subsequent studies.

## Conclusion

In conclusion, we screened key m6A regulatory genes, i.e., YTHDC1, RBM15, HNRNPA2B1, YTHDF3, and ALKBH5 based on RNA expression assay data from IVDD samples in the GEO database using mechanistic learning combined with LASSO regression analysis. Based on the above genes, we divided IVDD patients into two clusters to reveal the immune infiltration of different clusters and eventually identified the immune biomarker of IVDD, YTHDC1. Through functional enrichment, protein interaction network analysis, and molecular prediction analysis, we eventually discovered that YTHDC1 may affect the immune microenvironment of IVDD through ABL1 and TXK, which in turn influences the progression of IVDD.

### Supplementary Information

Supplementary Information.

## Data Availability

The original contributions presented in the study are included in the article, further inquiries can be directed to the corresponding author/s.

## References

[1] Choi YS (2009). Pathophysiology of degenerative disc disease. Asian Spine J..

[2] Maher C, Underwood M, Buchbinder R (2017). Non-specific low back pain. Lancet.

[3] Zhang Y, Sun Z, Liu J, Guo X (2008). Advances in susceptibility genetics of intervertebral degenerative disc disease. Int J Biol Sci..

[4] Tabor HK, Risch NJ, Myers RM (2002). Candidate-gene approaches for studying complex genetic traits: Practical considerations. Nat Rev Genet..

[5] Jonkhout N, Tran J, Smith MA, Schonrock N, Mattick JS, Novoa EM (2017). The RNA modification landscape in human disease. RNA.

[6] Liu ZX, Li LM, Sun HL, Liu SM (2018). Link between m6A modification and cancers. Front Bioeng. Biotechnol..

[7] Roignant JY, Soller M (2017). m6A in mRNA: An ancient mechanism for fine-tuning gene expression. Trends Genet..

[8] Gruber HE, Hoelscher GL, Ingram JA, Hanley EN (2012). Genome-wide analysis of pain-, nerve- and neurotrophin -related gene expression in the degenerating human annulus. Mol Pain..

[9] Gruber HE, Hoelscher G, Loeffler B, Chow Y, Ingram JA, Halligan W, Hanley EN (2009). Prostaglandin E1 and misoprostol increase epidermal growth factor production in 3D-cultured human annulus cells. Spine J..

[10] Wang Y, Dai G, Li L, Liu L, Jiang L, Li S, Liao S, Wang F, Du W, Li Y (2019). Transcriptome signatures reveal candidate key genes in the whole blood of patients with lumbar disc prolapse. Exp. Ther. Med..

[11] Tam V, Chen P, Yee A, Solis N, Klein T, Kudelko M, Sharma R, Chan WC, Overall CM, Haglund L, Sham PC, Cheah KSE, Chan D (2020). DIPPER, a spatiotemporal proteomics atlas of human intervertebral discs for exploring ageing and degeneration dynamics. Elife.

[12] Hu R, He K, Chen B, Chen Y, Zhang J, Wu X, Shi M, Wu L, Ma R (2024). Electroacupuncture promotes the repair of the damaged spinal cord in mice by mediating neurocan-perineuronal net. CNS Neurosci. Ther..

[13] Tang T, He Z, Zhu Z, Wang F, Chen H, Zhang F, Zhou J, Wang J, Li B, Liu X, Zhou Z, Liu S (2023). Identification of novel gene signatures and immune cell infiltration in intervertebral disc degeneration using bioinformatics analysis. Front Mol. Biosci..

[14] Han J, Kong H, Wang X, Zhang XA (2022). Novel insights into the interaction between N6-methyladenosine methylation and noncoding RNAs in musculoskeletal disorders. Cell Prolif..

[15] Xia J, Xie Z, Niu G, Lu Z, Wang Z, Xing Y, Ren J, Hu Z, Hong R, Cao Z, Han S, Chu Y, Liu R, Ke C (2023). Single-cell landscape and clinical outcomes of infiltrating B cells in colorectal cancer. Immunology.

[16] Su W, Wei Y, Huang B, Ji J (2021). Identification of hub genes and immune infiltration in psoriasis by bioinformatics method. Front Genet..

[17] Sun J, Yang F, Wang L, Yu H, Yang Z, Wei J, Vasilev K, Zhang X, Liu X, Zhao Y (2022). Delivery of coenzyme Q10 loaded micelle targets mitochondrial ROS and enhances efficiency of mesenchymal stem cell therapy in intervertebral disc degeneration. Bioact. Mater..

[18] Jutberger H, Lorentzon M, Barrett-Connor E, Johansson H, Kanis JA, Ljunggren O, Karlsson MK, Rosengren BE, Redlund-Johnell I, Orwoll E, Ohlsson C, Mellström D (2010). Smoking predicts incident fractures in elderly men: Mr OS Sweden. J. Bone Miner Res..

[19] Chuang YH, Ou HY, Yu CY, Chen CL, Weng CC, Tsang LL, Hsu HW, Lim WX, Huang TL, Cheng YF (2019). Diffusion-weighted imaging for identifying patients at high risk of tumor recurrence following liver transplantation. Cancer Imaging.

[20] Wang L, He T, Liu J, Tai J, Wang B, Zhang L, Quan Z (2021). Revealing the immune infiltration landscape and identifying diagnostic biomarkers for lumbar disc herniation. Front Immunol..

[21] Szklarczyk D, Gable AL, Nastou KC, Lyon D, Kirsch R, Pyysalo S, Doncheva NT, Legeay M, Fang T, Bork P, Jensen LJ, von Mering C (2021). The STRING database in 2021: Customizable protein-protein networks, and functional characterization of user-uploaded gene/measurement sets. Nucleic Acids Res..

[22] Kuhn M, Szklarczyk D, Franceschini A, Campillos M, von Mering C, Jensen LJ, Beyer A, Bork P (2010). STITCH 2: An interaction network database for small molecules and proteins. Nucleic Acids Res..

[23] David CJ, Chen M, Assanah M, Canoll P, Manley JL (2010). HnRNP proteins controlled by c-Myc deregulate pyruvate kinase mRNA splicing in cancer. Nature.

[24] Santarosa M, Del Col L, Viel A, Bivi N, D'Ambrosio C, Scaloni A, Tell G, Maestro R (2010). BRCA1 modulates the expression of hnRNPA2B1 and KHSRP. Cell Cycle.

[25] Kanehisa M, Goto S (2000). KEGG: Kyoto encyclopedia of genes and genomes. Nucleic Acids Res..

[26] Kanehisa M (2019). Toward understanding the origin and evolution of cellular organisms. Protein Sci..

[27] Kanehisa M, Furumichi M, Sato Y, Kawashima M, Ishiguro-Watanabe M (2023). KEGG for taxonomy-based analysis of pathways and genomes. Nucleic Acids Res..

[28] Kawaguchi Y (2018). Genetic background of degenerative disc disease in the lumbar spine. Spine Surg. Relat. Res..

[29] Martirosyan NL, Patel AA, Carotenuto A, Kalani MY, Belykh E, Walker CT, Preul MC, Theodore N (2016). Genetic alterations in intervertebral disc disease. Front Surg..

[30] Ravichandran D, Pillai J, Krishnamurthy K (2022). Genetics of intervertebral disc disease: A review. Clin. Anat..

[31] Tian Y, Yang J, Lan M, Zou T (2020). Construction and analysis of a joint diagnosis model of random forest and artificial neural network for heart failure. Aging (Albany NY).

[32] Doraisamy R, Ramaswami K, Shanmugam J, Subramanian R, Sivashankaran B (2021). Genetic risk factors for lumbar disc disease. Clin Anat..

[33] Nagasawa CK, Garcia-Blanco MA (2023). Early splicing complexes and human disease. Int. J. Mol. Sci..

[34] Zhang L, Li X, Kong X, Jin H, Han Y, Xie Y (2020). Effects of the NF-κB/p53 signaling pathway on intervertebral disc nucleus pulposus degeneration. Mol. Med. Rep..

[35] Liu Y, Qu Y, Liu L, Zhao H, Ma H, Si M, Cheng L, Nie L (2019). PPAR-γ agonist pioglitazone protects against IL-17 induced intervertebral disc inflammation and degeneration via suppression of NF-κB signaling pathway. Int. Immunopharmacol..

[36] Sun J, Tan Y, Su J, Mikhail H, Pavel V, Deng Z, Li Y (2023). Role and molecular mechanism of ghrelin in degenerative musculoskeletal disorders. J. Cell Mol. Med..

[37] Lin J, Du J, Wu X, Xu C, Liu J, Jiang L, Cheng X, Ge G, Chen L, Pang Q, Geng D, Mao H (2021). SIRT3 mitigates intervertebral disc degeneration by delaying oxidative stress-induced senescence of nucleus pulposus cells. J. Cell Physiol..

[38] Kaneyama S, Nishida K, Takada T, Suzuki T, Shimomura T, Maeno K, Kurosaka M, Doita M (2008). Fas ligand expression on human nucleus pulposus cells decreases with disc degeneration processes. J. Orthop. Sci..

[39] Calle ML, Urrea V, Boulesteix AL, Malats N (2011). AUC-RF: A new strategy for genomic profiling with random forest. Hum. Hered..

[40] Hur S, Min JY, Yoo J, Kim K, Chung CR, Dykes PC, Cha WC (2021). Development and validation of unplanned extubation prediction models using intensive care unit data: Retrospective, comparative, machine learning study. J Med Internet Res..

[41] Sarica A, Cerasa A, Quattrone A (2017). Random forest algorithm for the classification of neuroimaging data in alzheimer's disease: A systematic review. Front Aging Neurosci..

[42] Chen W, Tang D, Ou M, Dai Y (2020). Mining prognostic biomarkers of hepatocellular carcinoma based on immune-associated genes. DNA Cell Biol..

[43] Rabar S, Lau R, O'Flynn N, Li L, Barry P, Guideline Development Group (2012). Risk assessment of fragility fractures: Summary of NICE guidance. BMJ.

[44] Harrell FE, Lee KL, Mark DB (1996). Multivariable prognostic models: Issues in developing models, evaluating assumptions and adequacy, and measuring and reducing errors. Stat. Med..

[45] Moons KG, Kengne AP, Grobbee DE, Royston P, Vergouwe Y, Altman DG, Woodward M (2012). Risk prediction models: II. External validation, model updating, and impact assessment. Heart.

[46] Wang S, Tian S, Li Y, Zhan N, Guo Y, Liu Y, Xu J, Ma Y, Zhang S, Song S, Geng W, Xia H, Ma P, Wang X, Liao T, Duan Y, Jin Y, Dong W (2020). Development and validation of a novel scoring system developed from a nomogram to identify malignant pleural effusion. EBioMedicine..

[47] Moons KG, de Groot JA, Bouwmeester W, Vergouwe Y, Mallett S, Altman DG, Reitsma JB, Collins GS (2014). Critical appraisal and data extraction for systematic reviews of prediction modelling studies: The CHARMS checklist. PLoS Med..

[48] Royston P, Altman DG (2013). External validation of a Cox prognostic model: Principles and methods. BMC Med. Res. Methodol..

[49] Moons KG, Kengne AP, Woodward M, Royston P, Vergouwe Y, Altman DG, Grobbee DE (2012). Risk prediction models: I. Development, internal validation, and assessing the incremental value of a new (bio)marker. Heart.

[50] Vickers AJ, Holland F (2021). Decision curve analysis to evaluate the clinical benefit of prediction models. Spine J..

[51] Khan M, Lin J, Wang B, Chen C, Huang Z, Tian Y, Yuan Y, Bu J (2022). A novel necroptosis-related gene index for predicting prognosis and a cold tumor immune microenvironment in stomach adenocarcinoma. Front Immunol..

[52] Patil DP, Chen CK, Pickering BF, Chow A, Jackson C, Guttman M, Jaffrey SR (2016). m(6)A RNA methylation promotes XIST-mediated transcriptional repression. Nature.

[53] Wang T, Bai J, Zhang Y, Xue Y, Peng Q (2022). N6-Methyladenosine regulator RBM15B acts as an independent prognostic biomarker and its clinical significance in uveal melanoma. Front Immunol..

[54] Zhao Z, Ju Q, Ji J, Li Y, Zhao Y (2022). N6-Methyladenosine methylation regulator RBM15 is a potential prognostic biomarker and promotes cell proliferation in pancreatic adenocarcinoma. Front Mol. Biosci..

[55] Duan Y, Yu C, Yan M, Ouyang Y, Ni S (2022). m6A Regulator-mediated RNA methylation modification patterns regulate the immune microenvironment in osteoarthritis. Front Genet..

[56] Roundtree IA, Luo GZ, Zhang Z, Wang X, Zhou T, Cui Y, Sha J, Huang X, Guerrero L, Xie P, He E, Shen B, He C (2017). YTHDC1 mediates nuclear export of N6-methyladenosine methylated mRNAs. Elife.

[57] Fang K, Qu H, Wang J, Tang D, Yan C, Ma J, Gao L (2021). Characterization of modification patterns, biological function, clinical implication, and immune microenvironment association of mA regulators in pancreatic cancer. Front Genet..

[58] Gu C, Shi X, Qiu W, Huang Z, Yu Y, Shen F, Chen Y, Pan X (2021). Comprehensive analysis of the prognostic role and mutational characteristics of m6A-related genes in lung squamous cell carcinoma. Front Cell Dev. Biol..

[59] Li Y, Zhang W, Dai Y, Chen K (2022). Identification and verification of IGFBP3 and YTHDC1 as biomarkers associated with immune infiltration and mitophagy in hypertrophic cardiomyopathy. Front Genet..

[60] Yona G, Dirks W, Rahman S (2009). Comparing algorithms for clustering of expression data: How to assess gene clusters. Methods Mol Biol..

[61] Ryznar R, Wong C, Onat E, Towne F, LaPorta A, Payton M (2022). Principal component analysis of salivary cytokines and hormones in the acute stress response. Front Psychiatry.

[62] Bermudez-Lekerika P, Crump KB, Tseranidou S, Nüesch A, Kanelis E, Alminnawi A, Baumgartner L, Muñoz-Moya E, Compte R, Gualdi F, Alexopoulos LG, Geris L, Wuertz-Kozak K, Le Maitre CL, Noailly J, Gantenbein B (2022). Immuno-Modulatory Effects of Intervertebral Disc Cells. Front Cell Dev Biol..

[63] Xu H, Li J, Fei Q, Jiang L (2023). Contribution of immune cells to intervertebral disc degeneration and the potential of immunotherapy. Connect Tissue Res..

[64] Zhang F, Cui D, Wang K, Cheng H, Zhai Y, Jiao W, Wang Z, Cui X, Yu H (2023). Identifification and validation of ferroptosis signatures and immune infifiltration characteristics associated with intervertebral disc degeneration. Front Genet..

[65] Luxton HJ, Simpson BS, Mills IG, Brindle NR, Ahmed Z, Stavrinides V, Heavey S, Stamm S, Whitaker HC (2019). The oncogene metadherin interacts with the known splicing proteins YTHDC1, Sam68 and T-STAR and plays a novel role in alternative mRNA splicing. Cancers (Basel).

[66] Wang X, Liu C, Zhang S, Yan H, Zhang L, Jiang A, Liu Y, Feng Y, Li D, Guo Y, Hu X, Lin Y, Bu P, Li D (2021). N6-methyladenosine modification of MALAT1 promotes metastasis via reshaping nuclear speckles. Dev Cell..

[67] Rehfeld A, Plass M, Krogh A, Friis-Hansen L (2013). Alterations in polyadenylation and its implications for endocrine disease. Front Endocrinol. (Lausanne).

[68] Chen L, Fu Y, Hu Z, Deng K, Song Z, Liu S, Li M, Ou X, Wu R, Liu M, Li R, Gao S, Cheng L, Chen S, Xu A (2022). Nuclear m6 A reader YTHDC1 suppresses proximal alternative polyadenylation sites by interfering with the 3' processing machinery. EMBO Rep..

[69] Xu F, Yao W, Xue Y, Sun Q, Cao C (2022). The oncogene ABL1 regulates the inflammatory response of innate immunity via mediating TRAF6 ubiquitination. Immunobiology.

[70] Sommers CL, Rabin RL, Grinberg A, Tsay HC, Farber J, Love PE (1999). A role for the Tec family tyrosine kinase Txk in T cell activation and thymocyte selection. J. Exp. Med..

[71] Kashiwakura J, Suzuki N, Nagafuchi H, Takeno M, Takeba Y, Shimoyama Y, Sakane T (1999). Txk, a nonreceptor tyrosine kinase of the Tec family, is expressed in T helper type 1 cells and regulates interferon gamma production in human T lymphocytes. J. Exp. Med..

