# Supplementary figures and images for: Experimental validation and comprehensive analysis of m6A methylation regulators in intervertebral disc degeneration subpopulation classification
Source: Sci Rep. 2024 Apr 10;14:8417. doi: 10.1038/s41598-024-58888-w (PMC11006851; doi:10.1038/s41598-024-58888-w)

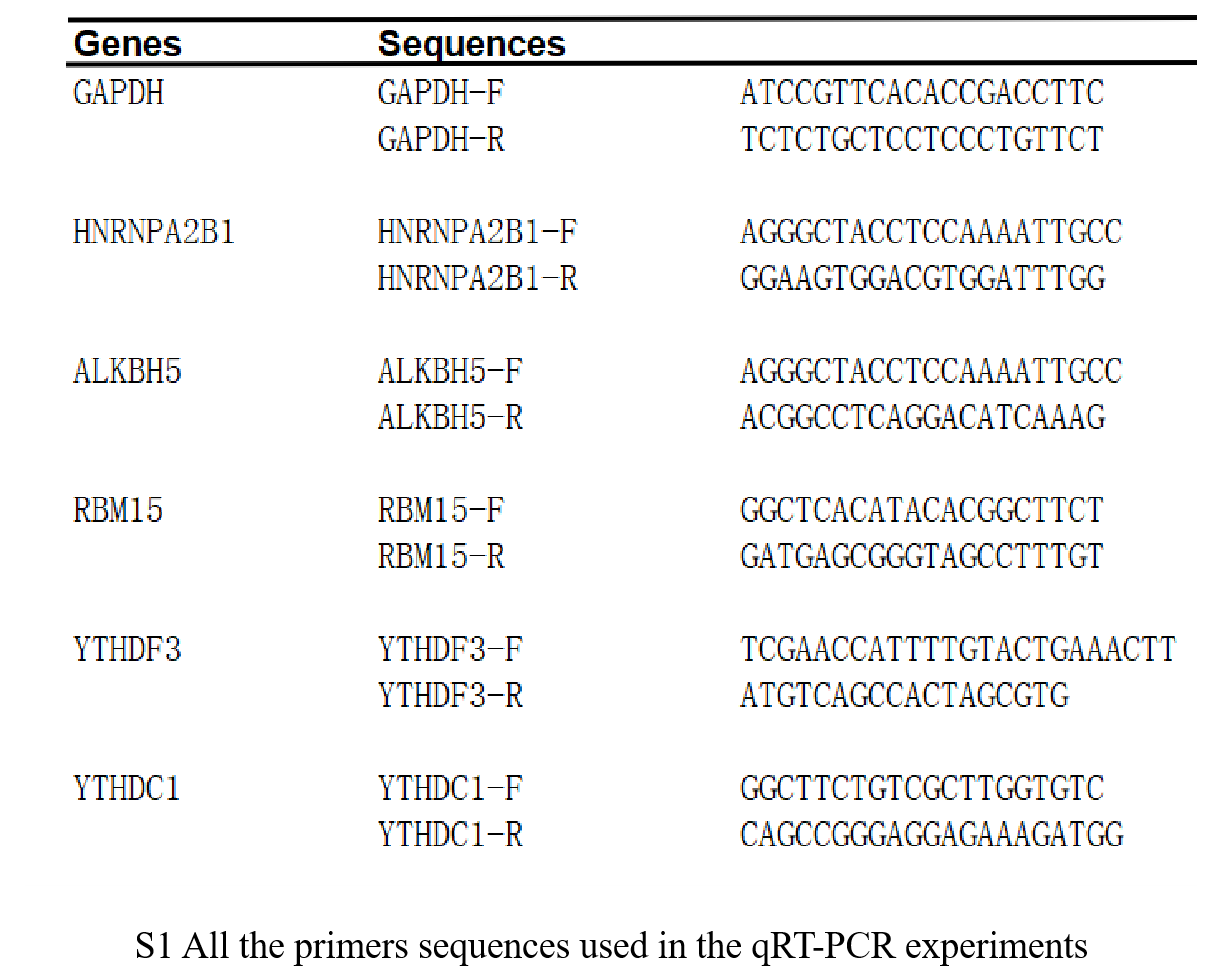

Supplement: Supplementary file 1 — Supplementary Information. [file 41598_2024_58888_MOESM1_ESM.tif]
